# Identification and application of exogenous dsRNA confers plant protection against *Sclerotinia sclerotiorum* and *Botrytis cinerea*

**DOI:** 10.1038/s41598-018-25434-4

**Published:** 2018-05-09

**Authors:** Austein G. McLoughlin, Nick Wytinck, Philip L. Walker, Ian J. Girard, Khalid Y. Rashid, Teresa de Kievit, W. G. Dilantha Fernando, Steve Whyard, Mark F. Belmonte

**Affiliations:** 1 Department of Biological Sciences, University of Manitoba, Winnipeg, Manitoba R3T 2N2 Canada; 2 Morden Research and Development Centre, Agriculture and Agri-Food Canada, Morden, Manitoba R6M 1Y5 Canada; 3 Department of Microbiology, University of Manitoba, Winnipeg, Manitoba R3T 2N2 Canada; 4 Department of Plant Science, University of Manitoba, Winnipeg, Manitoba R3T 2N2 Canada

## Abstract

*Sclerotinia sclerotiorum*, the causal agent of white stem rot, is responsible for significant losses in crop yields around the globe. While our understanding of *S*. *sclerotiorum* infection is becoming clearer, genetic control of the pathogen has been elusive and effective control of pathogen colonization using traditional broad-spectrum agro-chemical protocols are less effective than desired. In the current study, we developed species-specific RNA interference-based control treatments capable of reducing fungal infection. Development of a target identification pipeline using global RNA sequencing data for selection and application of double stranded RNA (dsRNA) molecules identified single gene targets of the fungus. Using this approach, we demonstrate the utility of this technology through foliar applications of dsRNAs to the leaf surface that significantly decreased fungal infection and *S*. *sclerotiorum* disease symptoms. Select target gene homologs were also tested in the closely related species, *Botrytis cinerea*, reducing lesion size and providing compelling evidence of the adaptability and flexibility of this technology in protecting plants against devastating fungal pathogens.

## Introduction

*Sclerotinia sclerotiorum* is a necrotrophic fungal pathogen and the causal agent of white stem rot in canola (*Brassica napus*). This ascomycete infects over 500 different plant species and causes major economic losses globally^1,2^. Current methods to control *S*. *sclerotiorum* infection involve the application of broad-spectrum fungicides, which have been attributed to the rise of chemical resistance and may have deleterious or unwanted effects on the surrounding agro-ecological landscape, if managed poorly^3–5^. Other practices, such as crop rotations, can be ineffective due to the formation of overwintering structures, termed sclerotia, which can remain in the soil for several years^6^. In addition, few plant cultivars are considered genetically resistant to *S*. *sclerotiorum* and further complicate effective disease management^7^. For these reasons, new, environmentally-safe, species-specific fungicides capable of suppressing *S*. *sclerotiorum* are needed.

Species-specific molecular insecticides, using RNA interference (RNAi) approaches, have been shown to control insect pests in the laboratory^8–11^, and recently, the first transgenic plants with RNAi genetic constructs have been approved for field use^12^. RNAi technologies are dependent on double stranded RNA (dsRNA) molecules, which are designed with complementary sequences to a given mRNA within the target organism. Once the dsRNA molecules enter the cell, they complex with DICER and the molecule is fragmented into small interfering RNAs (siRNAs), 21–24 nucleotides in length. The siRNAs then associate with ARGONAUTE, forming the RNA-induced silencing complex (RISC), which acts as an endonuclease to cleave mRNA molecules that share complementarity with the internalized siRNA sequences^13–15^. While the number of studies that describe the application of RNAi to control insect pests is increasing steadily, there are considerably fewer studies that describe the potential of RNAi to control fungal plant pathogens, despite the characterization of RNAi machinery in different fungal species^16–19^.

Recently, *S*. *sclerotiorum* engineered to express an RNAi construct targeting *SsITL* (integrin-like immune suppressor), *SsMADS* (MADS transcription factor), *SsSl2* (a cell wall protein), and *SsBi1* (Bax-inhibitor protein) showed compromised pathogenicity and altered cellular development^20–23^. Another study demonstrated limited fungal lesion formation on tobacco plants (*Nicotiana tabacum*) expressing hairpin RNA (hpRNA) molecules targeting *S*. *sclerotiorum* chitin synthase^24^. Despite these advances promoting the effectiveness of RNAi, the ability to control *S*. *sclerotiorum* is still limited and no study has shown effective control of this fungus on the leaf surface using topical applications of dsRNA molecules.

However, a pioneering study demonstrated the potency of synthesized dsRNA molecules under *in vitro* conditions. Molecules were designed to target essential genes within *Fusarium oxysporum* f. sp. *cubense* and *Mycosphaerella fijiensis*^25^. DsRNAs have also been used to control *Botrytis cinerea* and *Fusarium graminearum* infections *in planta*. DsRNAs that targeted Dicer-like 1 and 2 (Dcl1 and 2) transcripts in *B*. *cinerea*, reduced disease symptoms in a range of plant tissues^16^. Similarly, dsRNA targeting three *F*. *graminearum* cytochrome P450 lanosterol C-14-α demethylases protected barley (*Hordeum vulgare* L.) against fungal colonization^17^. These studies established the feasibility of topical applications of dsRNA to control pathogenic fungi. However, in both studies, the researchers demonstrated that targeting more than one protein-encoding mRNA was more effective than targeting a single transcript, despite successful implementations transgenically^24,26^. To date, no study has demonstrated effective fungal foliar suppression targeting only a single transcript, or has identified multiple unique targets for RNAi-based fungal management.

In this study, RNA sequencing (RNA-seq) was used to identify genes associated with fungal pathogenicity in the *B*. *napus – S*. *sclerotiorum* pathosystem and to uncover targets for RNAi. Bioinformatics analysis identified global changes of *S*. *sclerotiorum* gene expression on both susceptible (*B*. *napus cv*. *Westar*) and moderately tolerant (*B*. *napus cv*. *ZhongYou821* (*ZY821*)) cultivars, revealing biological processes associated with growth, cellular homeostasis, and infection. DsRNA molecules were designed to target genes associated with reactive oxygen species (ROS) responses, transcription, and host colonization, as well as those identified as essential in *Aspergillus fumatigus*^27^. Target transcripts were knocked down *in vitro* and topical applications of dsRNA reduced lesion progression on *B*. *napus* leaves successfully. Knockdown of many of these target mRNAs also proved effective in suppressing *S*. *sclerotiorum* growth on leaves of *Arabidopsis thaliana*. The versatility of the RNAi targets was also demonstrated by reducing the growth of another phytopathogenic fungus, *B*. *cinerea*. Taken together, the results demonstrate the utility of RNA-seq technology to guide the selection of multiple target genes for RNAi and to extend the utility of large scale datasets to protect agronomically-important plants against phytopathogenic fungi.

## Results

### Gene expression of *S*. *sclerotiorum* grown *in vitro* and on susceptible and tolerant hosts of *B*. *napus*

**Figure 1:**
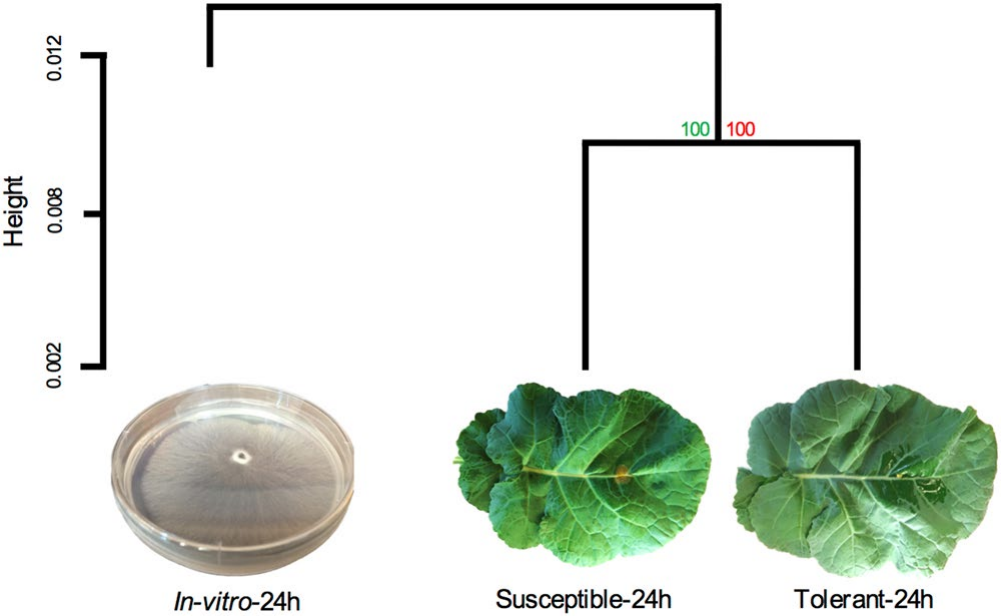
Growth and penetration of *S*. *sclerotiorum in-vitro* (PDA) and *in-planta* (*Brassica napus* cv. Westar (Susceptible) and ZhongYou821 (Tolerant)) with a hierarchical clustering analysis of global gene activity based on FPKM transcript abundances and a minimum detection value of 1 FPKM. Approximately unbiased (au) values found in green and bootstrapping p-values (bp) in red. Height represents correlation value between sub-branches.

Next generation RNA sequencing was used to identify similarities and differences in gene expression between *in vitro* plate-grown cultures and *in planta*-grown *S*. *sclerotiorum* (Table S1). Hierarchical clustering of FPKM (fragments per kilobase of transcript per million mapped reads) values revealed *S*. *sclerotiorum* grown *in vitro* was transcriptomically distinct from cultures grown for 24 hours on *B*. *napus* leaves (Fig. 1). Specifically, *S*. *sclerotiorum* grown on either tolerant (cv. ZY821) or susceptible (cv. Westar) plants clustered together with a bootstrapping score of 100, indicating that the expression of many *S*. *sclerotiorum* genes was dependent on the nature of the nutrition source, either the nutrient-accessible *in vitro* culture or the more complex host canola cultivars.

### Differential expression and GO term enrichment analysis in target gene identification

**Figure 2:**
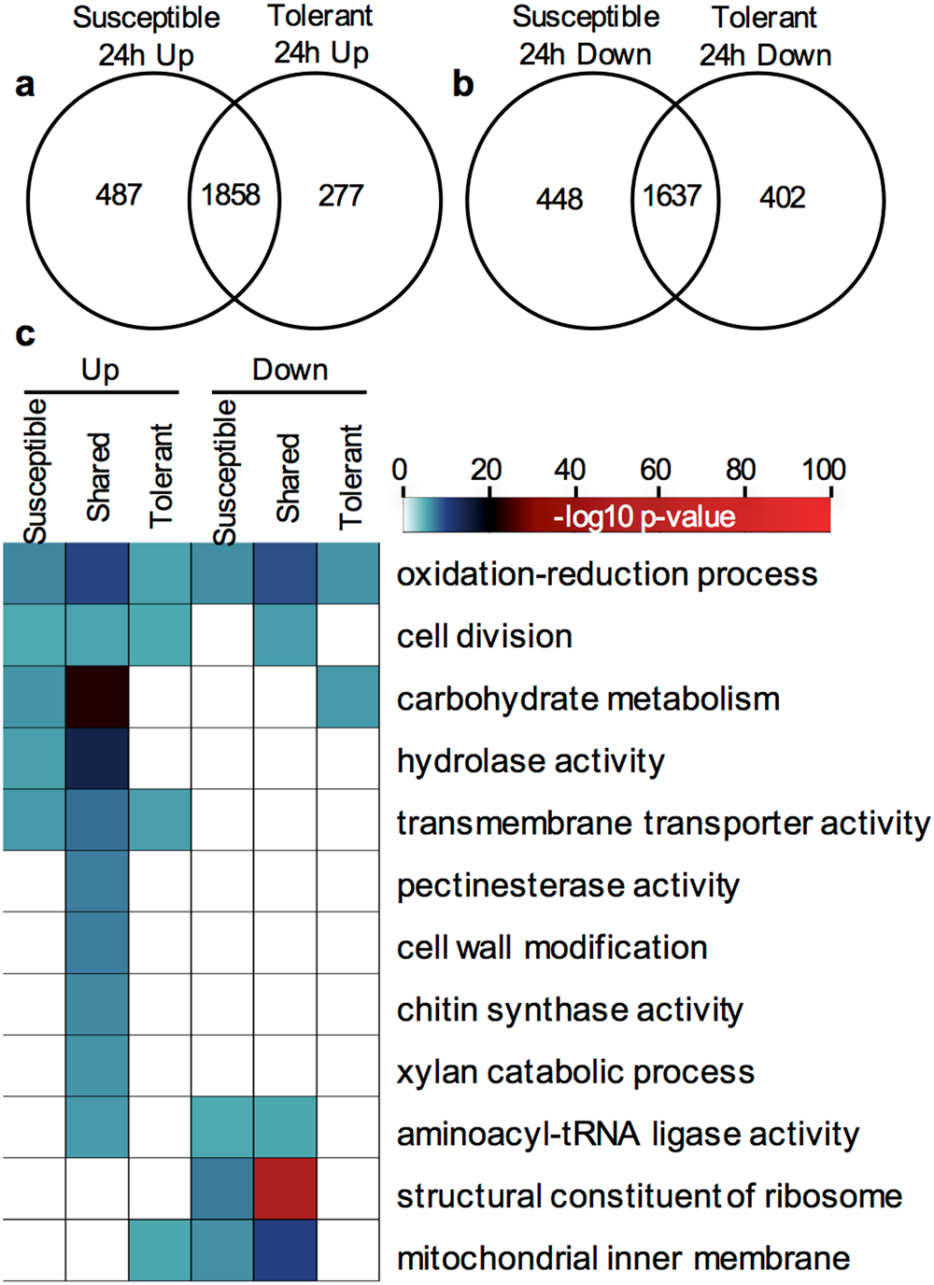
Identification of *S*. *sclerotiorum* genes and biological processes significantly up and down regulated during infection (*in-planta*). (**a**) Venn diagram showing up-regulated genes in *S*. *sclerotiorum* 24 hpi on susceptible (*Westar*) and tolerant (*ZY821*) genotypes of *B*. *napus*. (**c**) Venn diagram showing down-regulated genes in *S*. *sclerotiorum* 24 hpi on susceptible (*Westar*) and tolerant (*ZY821*) genotypes of *B*. *napus*. (**c**) Heatmap of enriched GO terms associated with significantly up and down regulated genes *in-planta* grown *S*. *sclerotiorum* during infection. GO terms are considered statistically significant if the hypergeometric p-value < 0.05.

The hierarchical clustering of *S*. *sclerotiorum* gene expression in the three transcriptomes likely reflects differences in how the fungus responds to and infects different plant cultivars. To identify genes that may be responsible for the plant-infection process, a comparison of global gene expression of *S*. *sclerotiorum* grown on susceptible and tolerant leaves of *B*. *napus* to those grown *in vitro* was performed. The analyses identified 1,858 genes that were significantly up and 1,637 that were significantly down-regulated in both Westar and ZY821, relative to the *in vitro*-growth fungus (Fig. 2a,b). Differential expression was also observed between the susceptible Westar and partially resistant ZY821 cultivars of *B*. *napus*, with 487 and 448 genes being up and down-regulated, respectively, in the Westar cultivar, and 277 and 402 genes being up and down-regulated in the ZY821 cultivar, respectively.

A gene ontology term enrichment analysis was performed on the significantly up and down-regulated genes to identify biological processes associated with pathogenesis. Processes that showed conserved enrichment *in-planta* and *in-vitro* included *oxidation-reduction processes*, *mycelium development*, and *cell division* (Fig. 2c). Significant enrichment of several processes involved in the host-pathogen interaction *in planta*, relative to *in vitro*-grown fungus, were also observed, such as: *carbohydrate metabolic processes*, *hydrolase activity*, and *transmembrane transport*. In contrast, processes that were down-regulated during *in-planta* growth included: protein synthesis and energy production. Additionally, some enriched biological processes such as *carbohydrate metabolism*, *hydrolase activity*, and *transcription factor activity* were differentially expressed during colonization on the susceptible and moderately tolerant cultivars of *B*. *napus*. Complete lists of significantly differentially expressed genes and their respective FPKM values, as well as significantly enriched GO terms and their respective p-values can be found in Additional file 1.

### QRT-PCR assessment of RNAi in *S*. *sclerotiorum*

**Figure 3:**
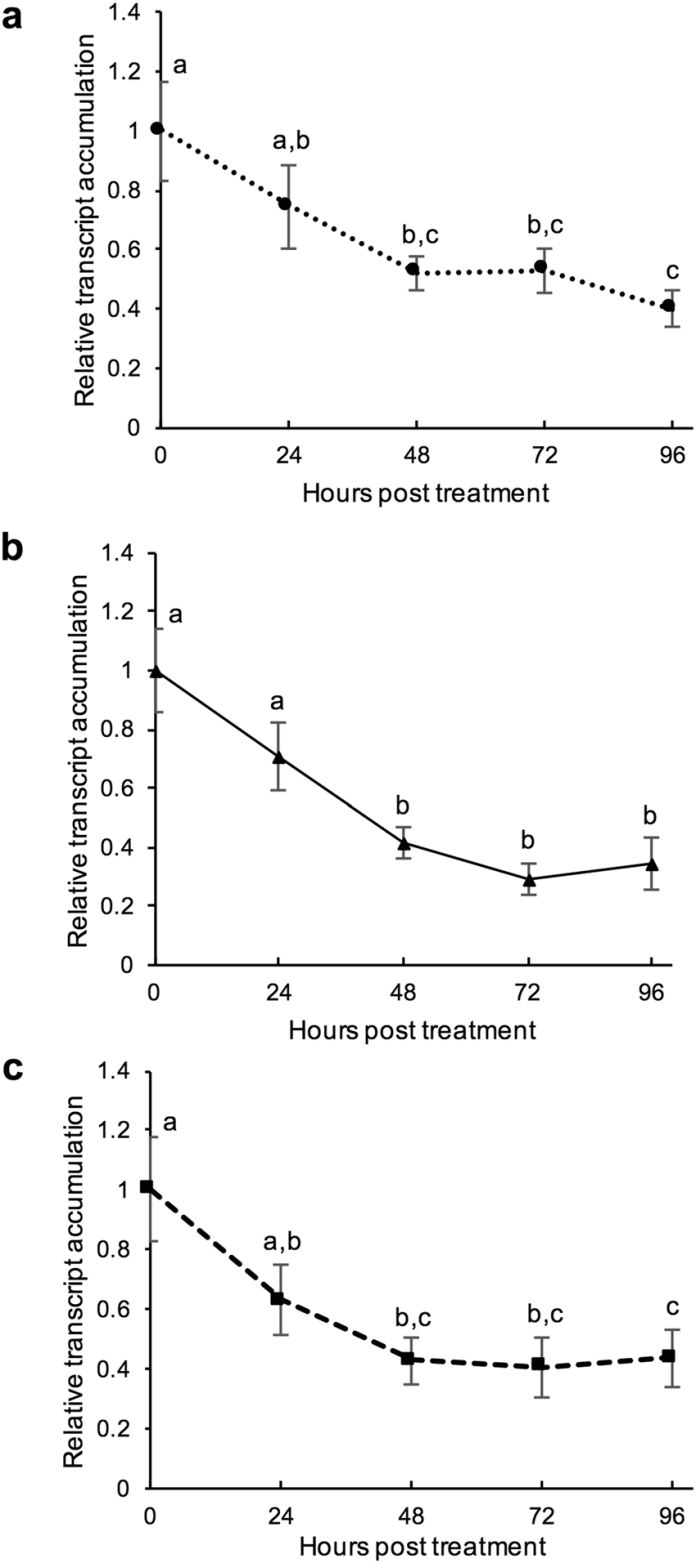
The timing of RNAi silencing in *Sclerotinia sclerotiorum in vitro*. Transcript levels were measured at time points 0, 24, 48, 72, and 96 hours post treatment of 500 ng/mL of dsRNA targeting SS1G_01703 (**a**), SS1G_05899 (**b**), SS1G_06487 (**c**), or GFP in liquid culture. Data are relative to GFP-dsRNA control and normalized to reference *SsSac7* (SS1G_12350). Data represents 3 biological replicates with error bars representing standard error. To test effect of timing, a one-way ANOVA (with significance of p < 0.05) was performed and followed by a Tukey post hoc test to compare means, where significant differences (p_tukey_ < 0.05) are denoted with differing letters.

To assess the duration of dsRNA mediated gene knockdown, *S*. *sclerotiorum* was grown in liquid cultures containing dsRNA molecules targeting the following three genes: SS1G_01703, amino acyl tRNA ligase; SS1G_05899, thioredoxin reductase; and SS1G_06487, TIM44. The dsRNAs were separately co-incubated with *S*. *sclerotiorum* at 500 ng/mL and transcript expression was assessed by qRT-PCR at 0, 24, 48, 72, and 96 hours post inoculation (hpi) (Fig. 3). The transcript levels of the three genes did not significantly differ from 0 hpi to 24 hpi (one-way analysis of variance (ANOVA) with Tukey post-hoc test, p_tukey_ = 0.325 (SS1G_01703, Fig. 3a), 0.282 (SS1G_05899, Fig. 3b), 0.115 (SS1G_06487, Fig. 3c)). By 48 hpi, all three genes’ transcripts were reduced significantly by 48–59% compared to 0 hpi (p_tukey_ = 0.022, (SS1G_01703); 0.001 (SS1G_05899); 0.005 (SS1G_06487). The level of suppression persisted for 96 hpi, not significantly changing from 48 hpi for the target genes tested (48 hpi vs. 96 hpi; p_tukey_ = 0.506, (SS1G_01703); 0.732 (SS1G_05899); 0.475 (SS1G_06487)). Therefore, the results indicate that 48 hours is required for optimal RNAi silencing to occur within *S*. *sclerotiorum* using topical dsRNA.

**Figure 4:**
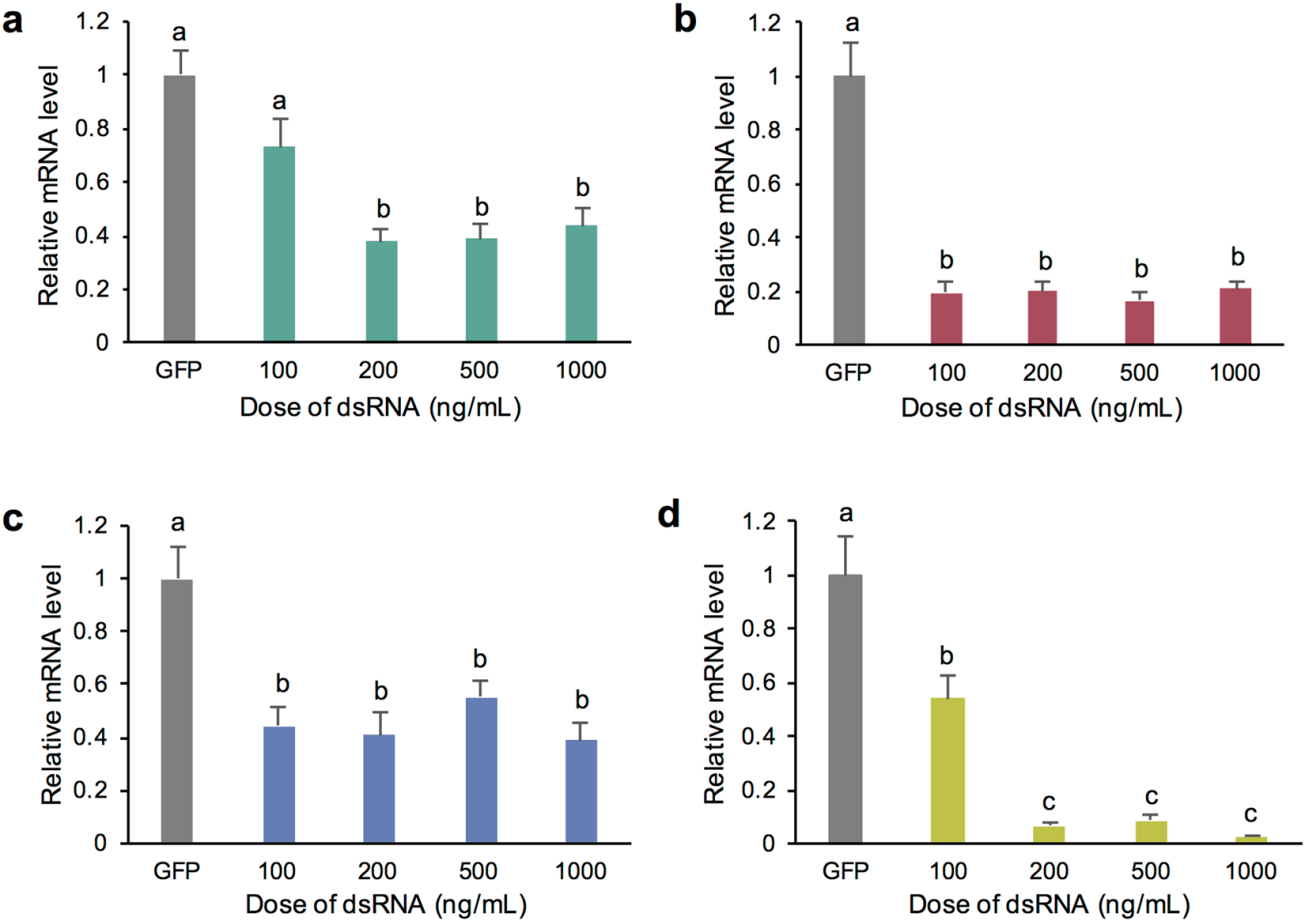
The effect of dsRNA dose on transcript levels in *Sclerotinia sclerotiorum in vitro*. Transcript levels were measured at time points 0, 24, 48, 72, and 96 hours post treatment of 500 ng/mL of dsRNA targeting SS1G_01703 (**a)**, SS1G_05899 (**b)**, SS1G_06487 (**c)**, SS1G_11912 (**d)**, or GFP in liquid culture. Data are relative to GFP-dsRNA control and normalized to reference *SsSac7* (SS1G_12350). Data represents 3 biological replicates with error bars representing standard error. To test effect of dosing, a one-way ANOVA (with significance level of p < 0.05) was performed and followed by a Tukey post hoc test to compare means, where significant differences (p_tukey_ < 0.05) are denoted with differing letters.

To examine dsRNA dose effects on target gene knockdown, liquid cultures of *S*. *sclerotiorum* were exposed to a range of doses (100–1000 ng/mL) of dsRNAs targeting the previously mentioned three genes and one additional gene that showed upregulation *in planta*; SS1G_11912, necrosis/ethylene-inducing peptide 2 (Fig. 4). The dsRNA targeting SS1G_05899 showed a 79–85% reduction in transcript abundance across all doses tested compared to the GFP control (one-way ANOVA with Tukey Post-hoc test, p_tukey_ < 0.001 (all doses); Fig. 4b). Similarly, SS1G_06487 showed a 45–60% reduction in transcript accumulation compared to GFP-dsRNA treated fungus (one-way ANOVA with Tukey Post-hoc test; p_tukey_ = 0.008 (100 ng/mL), 0.004 (200 ng/mL), 0.049 (500 ng/mL), 0.003 (1000 ng/mL); Fig. 4c). However, for the SS1G_01703-dsRNA treatment, a dose of at least 200 ng/mL was required to elicit a significant reduction compared to the GFP control (one-way ANOVA with Tukey Post-hoc test; p_tukey_ = 0.0788 (100 ng/mL), 0.042 (200 ng/mL); Fig. 4a). Higher doses of dsRNA did not change the level of reduction significantly from 200 ng/mL (p_tukey_ = 1 (500 ng/mL), 0.99 (1000 ng/mL)). A varied dose response was also observed for SS1G_11912, which required a dose of at least 200 ng/mL to achieve a maximum reduction of 94% (one way ANOVA with Tukey Post-hoc test; p_tukey_ < 0.001 (GFP vs. 200 ng/mL), p_tukey_ = 0.001 (100 ng/mL vs. 200 ng/mL); Fig. 4d). Similarly, doses higher than 200 ng/mL did not differ in terms of silencing response (p_tukey_ = 0.958 (200 ng/mL vs. 500 ng/mL), 0.193 (200 ng/mL vs. 1000 ng/mL), 0.108 (500 ng/mL vs. 1000 ng/mL)). The data suggests differential doses were required to achieve a maximal knockdown amongst the targets. However, once optimal knockdown was achieved, higher doses did not elicit a stronger silencing response.

### Foliar applications of dsRNA reduce *S*. *sclerotiorum* infection in *B*. *napus*

**Figure 5:**
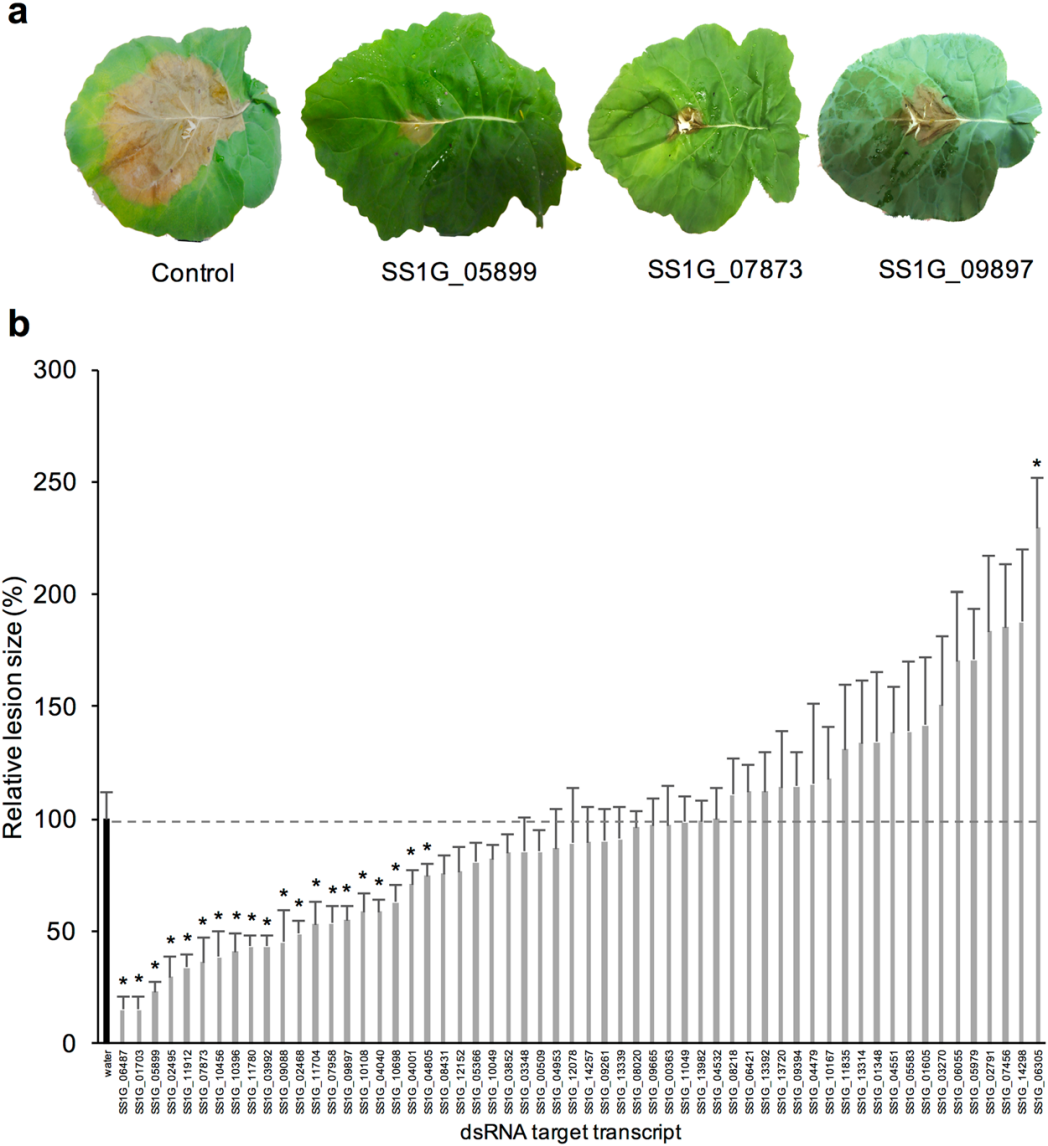
dsRNA targeting *S*. *sclerotiorum* suppresses lesion size on *B*. *napus* susceptible cultivar Westar (**a**) *S*. *sclerotiorum* infection lesions on *Brassica napus cv*. *Westar* following a treatment of 200 ng dsRNA targeting *S*. *sclerotiorum* genes at 2 dpi. (**b**) Average lesion size (n = 10 leaves) relative to control (black bar) with error bars corresponding to standard error. Significant difference from control represented by asterix (*)(student’s t-test with Bonferroni correction; p < 0.00083).

Having confirmed that dsRNAs could reduce transcript abundance in *S*. *sclerotiorum* for at least 96 hpi using relatively low concentrations, the level of protection imparted to *B*. *napus* plants was then assessed using a petal infection assay that facilitated aggressive *S*. *sclerotiorum* infection^28^. Leaf surfaces treated with water + Silwet L-77 resulted in rapid *S*. *sclerotiorum* infection that developed large necrotic lesions by 2 dpi (Figs 5a and S2). In contrast, when the leaf surface was treated with an application of *S*. *sclerotiorum*–specific dsRNAs + Silwet L-77, dramatic reductions in lesion size and morphology were observed for dsRNA treatments (Fig. 5a). Of the 59 dsRNAs tested, 20 showed a significant reduction in lesion size, ranging from 26 to 85% (*student’s t-test* with Bonferroni correction, p < 8 × 10^−4^; Fig. 5b and Table 1; Additional file 2). Of these 20 dsRNA molecule treatments, 18 conformed to the criteria outlined in the target identification pipeline (TIP; Table S2). Some of the dsRNA molecules nominated using the TIP selection criteria included genes involved in toxin biosynthesis (SS1G_01703), ROS response (SS1G_02495), and cell cycle regulation (SS1G_09897). Targeting these genes’ transcripts with dsRNA resulted in significant reductions in lesion size by 85%, 71%, and 45%, respectively. Similarly, *A*. *fumatigus* essential gene homologues associated with ribosomal biogenesis (SS1G_07873) and mitochondrial protein import (SS1G_06487) also significantly reduced fungal lesion formation by 64% and 85%, respectively (Fig. 5b and Table 1).

**Table 1:** Selected list of *S*. *sclerotiorum t*arget genes for RNA interference testing in liquid culture, and infection assays on *B*. *napus* and *A*. *thaliana*.

Source	Gene	FPKM *in vitro*	FPKM Westar 24 dpi	FPKM ZY821 24 hpi	Process
Literature^1^	SS1G_08218	7913.2	5211.0	6743.8	Oxaloacetate acetylhydrolase
Essential Genes^2^	SS1G_05899	268.7	259.1	244.4	Thioredoxin reductase
Essential genes^2^	SS1G_06487	87.3	82.0	114.3	TIM44
Essential genes^2^	SS1G_07873	47.1	41.5	47.8	pre-40S ribosomal particle
Low expression values	SS1G_00509	1.8	6.6	3.2	Hydrolase activity, carbohydrate metabolic process
Low expression values	SS1G_06055	0.0	0.8	2.0	Prenyltransferase activity
Low expression values	SS1G_13720	0.0	1.4	0.5	MFS sugar transporter
Low expression values	SS1G_13982	0.1	0.2	2.0	Triacylglycerol lipase
Moderate expression values	SS1G_09261	14.20	22.56	22.80	Bub1-Bub3 complex localization to kinetochore
High accumulation values	SS1G_10167	14126.6	19992.7	24179.3	Endo-polygalacturonase
Positive expression fold change^3^	SS1G_02791	2.0	19.1	23.8	Transcription factor activity
Positive expression fold change^3^	SS1G_04551	1.9	12.6	16.4	Pectinesterase
Positive expression fold change^3^	SS1G_14298	17.9	31.6	23.0	bHLH transcription factor
TIP^4^	SS1G_01703	45.1	307.0	164.7	Aminoacyl-tRNA ligase activity/alfatoxin biosynthesis
TIP^4^	SS1G_02495	61.2	336.1	353.7	Peroxidase activity
TIP^4^	SS1G_03208	11.5	51.4	48.3	Pre-mRNA splicing factor 8
TIP^4^	SS1G_03991	16.7	39.5	39.4	Srb8 component of mediator complex
TIP^4^	SS1G_04966	30.6	64.3	59.5	Histone modification
TIP^4^	SS1G_06830	13.7	101.8	71.3	Aminoacyl permease
TIP^4^	SS1G_09680	50.8	61.2	70.3	60 S ribosome biogenesis
TIP^4^	SS1G_09897	28.53	61.36	59.24	Cdc25
TIP^4^	SS1G_11912	24.5	551.2	359.3	Necrosis/ethylene inducing peptide 2
TIP^4^	SS1G_12021	149.3	397.4	363.8	1,3 glucan synthase
TIP^4^	SS1G_12640	53.1	114.2	130.1	Protein disulphide oxidoreductase process
TIP^4^	SS1G_12992	13.5	57.5	39.0	Transglutaminase protein modification in ER
TIP^4^	SS1G_13702	127.1	98.2	101.9	TIM23
TIP^4^	SS1G_13746	15.1	33.7	32.5	Peroxisome

(**1**) Liang *et al*.^29^; (**2**) Database of Essential Genes (www.essentialgene.org)^45^; (**3**) Positive fold change in response to *in planta* growth (**4**) TIP = Target identification pipeline (Table S2).

Interestingly, one of the dsRNA treatments targeting SS1G_06305, a probable transcription factor, increased lesion size 129% (p < 8 × 10^−4^) compared to the control treatment (Fig. 5b). Furthermore, 39 genes targeted by some dsRNA molecules caused no significant impact on fungal lesion sizes on the leaves; these dsRNAs targeted genes involved in a range of cellular processes, including carbohydrate catabolism (SS1G_00509; endo-1,4-beta-xylanase), hydrolysis (SS1G_13982; triacylglycerol lipase), and kinetochore functions (SS1G_09261). Even a previously characterized genetic deletion target^29^, SS1G_08218 (*Ssoah*; oxaloacetate acetylhydrolase), had no significant impact on disease symptoms under the conditions tested size (p = 0.5). While these targets did not show significant modulations in lesion size, they helped define criteria for TIP.

### Topical dsRNAs mitigate *S*. *sclerotiorum* infection on *A*. *thaliana*

**Figure 6:**
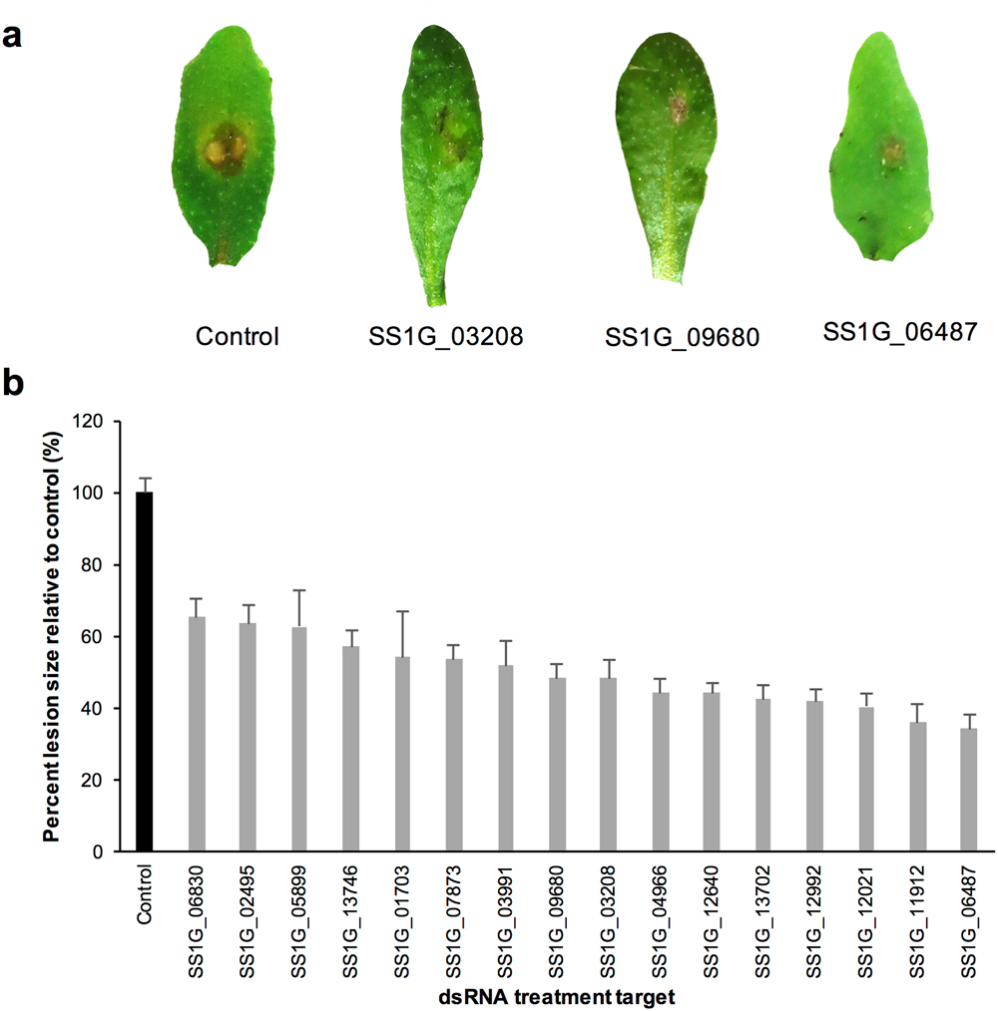
DsRNA treatment on *A*. *thalaina* leaves reduces *S*. *sclerotiorum* lesion area. (**a**) *S*. *sclerotiorum* spore inoculation on *A*. *thaliana* leaves after a foliar application 200 ng of specific dsRNA spread over the entire leaf surface at 4 dpi. (**b**) Average lesion relative to control (darker bar) with standard error bars of 3 bioreps of 10 leaves (n = 30). All targeting dsRNA treatment were significantly different from control (student’s t-test with Bonferroni correction, p < 0.0031).

Given the extensive host range of *S*. *sclerotiorum*, it was of interest to determine whether this fungus could be similarly suppressed using dsRNAs in another plant species. Using the model plant *A*. *thaliana*, 10 new *S*. *sclerotiorum* genes, nominated using TIP selection criteria (Tables 1 and S2), were assessed in parallel with 6 previously tested molecules used on *B*. *napus* (SS1G_01703, SS1G_02495, SS1G_05899, SS1G_06487, SS1G_07873, and SS1G_11912; Fig. 5b). Using an adapted spore inoculation technique for canola cotyledons, lesion sizes on *A*. *thaliana* leaves treated with 200 ng dsRNA were scored at 4 dpi^30^. Significant reductions in lesion size between 34–66% were observed by targeting genes associated with processes such as mRNA splicing (SS1G_03208), ribosome biogenesis (SS1G_09680), protein disulphide oxidoreductase (SS1G_12640), and a peroxisomal protein (SS1G_13746) (student’s t-test with Bonferroni correction, p < 0.0031; Fig. 6b).

DsRNA treatments on *B*. *napus* leaves also reduced infection on *A*. *thaliana* significantly, such as SS1G_11912, which reduced lesion sizes by 64–66% in both plant species. However, there were noticeable differences in efficacy of the other five dsRNAs, and did not correlate between lesion sizes on the plant species (R^2^ = 0.11, p = 0.8). Specifically, the dsRNAs targeting SS1G_02495 and SS1G_07873, which reduced lesions 71 and 64%, respectively in *B*. *napus*, only reduced lesions by 34 and 46% in *A*. *thaliana*, respectively. Overall, dsRNA treatments reduced the fungal progression on the spore-inoculated leaves significantly and still suggests that dsRNA used to protect one plant species could also be applied to protect another.

### DsRNAs targeting homologues in *Botrytis cinerea* attenuate fungal infection

**Figure 7:**
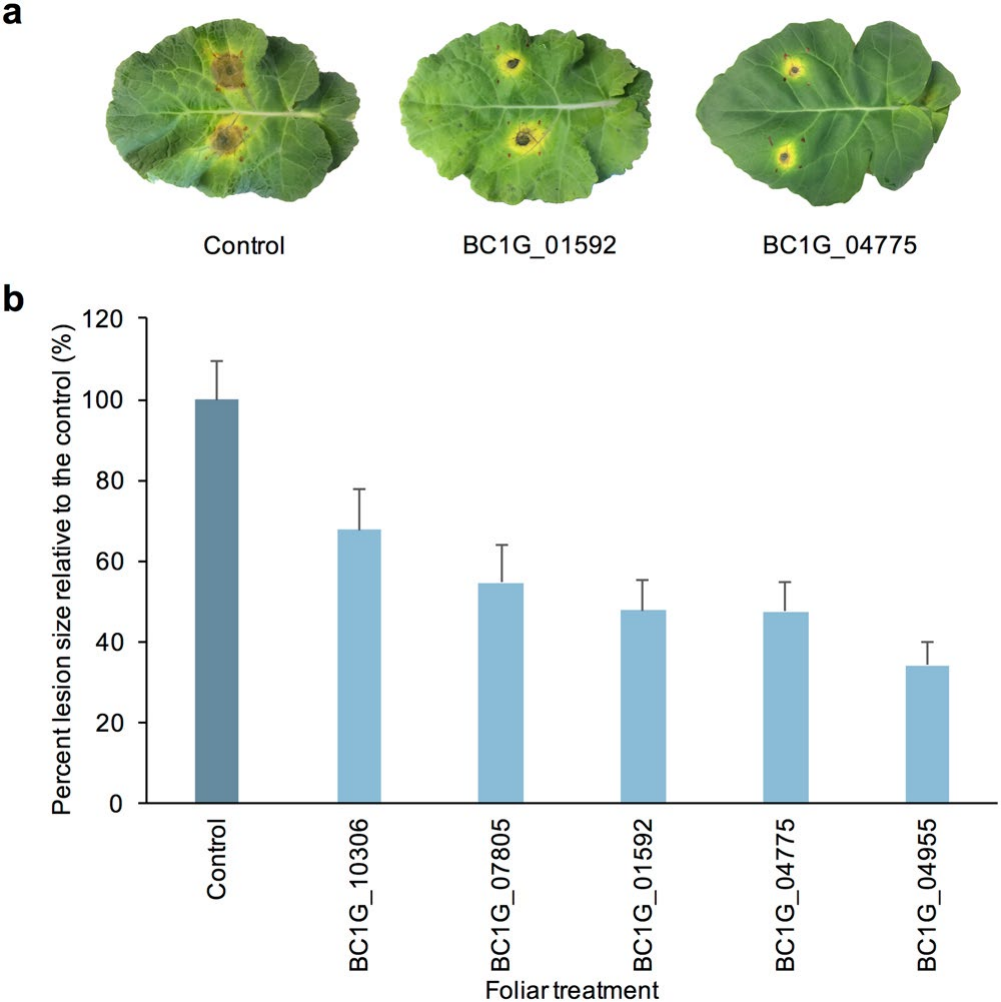
*Botrytis cinerea* homologues targeted with foliar dsRNA controls fungal infection (**a**) *B*. *cinerea* spore inoculation of *B*. *napus cv*. *Westar* leaves after an application of 500 ng of dsRNA targeting *B*. *cinerea* genes was spread over a 4 cm^2^ area at 4 dpi. (**b**) Average lesion size compared to control (darker bar) with 4 bioreps of 10 leaves each (n = 40). Error bars represent standard error. All targeting dsRNA treatment were significantly different from GFP control (student’s t-test with Bonferroni correction, p < 0.01).

*Botrytis cinerea* is a phytopathogenic fungus closely related to *S*. *sclerotiorum*. Hence, it was of interest to determine whether homologues of *S*. *sclerotiorum* target genes identified during earlier screens could be similarly used as RNAi targets to suppress *B*. *cinerea* infections on *B*. *napus*. *Botrytis cinerea-*specific dsRNA molecules were designed to target five homologues identified in *S*. *sclerotiorum* (Additional file 2). Using a detached leaf assay, mature *B*. *napus cv*. *Westar* leaves were coated with dsRNA, infected with *B*. *cinerea* spores, and scored for lesion size after 4 dpi. Leaves treated with dsRNA targeting BC1G_04775 (SS1G_06487 homologue) and BC1G_01592 (SS1G_05899 homologue) formed smaller brown necrotic regions than the control leaves (Fig. 7a). Treatments of dsRNA targeting BC1G_04955 (SS1G_02495 homologue), BC1G_04775, BC1G_01592, BC1G_07805 (SS1G_07873 homologue), and BC1G_10306 (SS1G_11912 homologue), on average, reduced lesion sizes by 66% (Fig. 7b; *student’s t-test* with Bonferroni correction; p < 0.01). Interestingly, the dsRNA targeting the SS1G_11912 homologue, BC1G_010306, showed only moderate reductions in lesion area, suggesting that the gene product may be more important for *S*. *sclerotiorum* infection rather than *B*. *cinerea*. Despite the overall reductions, the efficacies of dsRNAs targeting *S*. *sclerotiorum* and *B*. *cinerea* did not correlate (R^2^ = 0.39, p = 0.2). However, the reductions in both species suggests these targets could be used to control a related fungal species. A complete summary of foliar results can be found in Additional file 2.

## Discussion

Our transcriptome interrogation of the *B*. *napus*-*S*. *sclerotiorum* interface uncovered novel targets of fungal growth and pathogenicity that helped guide the development of an RNAi target identification pipeline (TIP; Table S2), thus providing a clear and rational link between transcript profiling and effective dsRNA molecule design. We demonstrated dsRNA-mediated *in-vitro* knockdown of *S*. *sclerotiorum* transcripts, and a foliar application of dsRNAs conferred significant protection to both *B*. *napus* and *A*. *thaliana* when challenged aggressively with the necrotrophic pathogen. Extension of this technology to homologous transcripts of *B*. *cinerea* revealed cross-species control of major fungal pathogens using topical applications of dsRNAs targeting a single gene.

When dsRNAs were incubated with *S*. *sclerotiorum in vitro*, transcript level reductions were observed for all genes tested, providing clear evidence of uptake and an RNAi response. Although the dsRNA uptake mechanism has yet to be established in fungi, fluorescein-labelled dsRNAs have been observed accumulating within *B*. *cinerea* spores^16^, and the results are consistent with other eukaryotic organisms that showed reductions in transcript levels within 48 hours^31–33^. The dose-response for individual genes varied, and high doses failed to elicit further transcript reductions (Fig. 4), as reflected in previous insect and flatworm research^31,34,35^. At higher dosing levels, the RNAi silencing machinery could have been saturated with molecules, and thus could have been unable to process the totality of the molecules at once. Furthermore, differences in knockdown response may be attributed to endogenous transcript levels and mRNA turnover rate, GC content, or other physical properties associated with dsRNA structure^36–39^. Recent developments in nucleotide modification and delivery methods promise to boost efficacy of RNAi^40–42^. However, without modification, our molecules guarded against virulent necrotrophic pathogens, putting RNAi at the forefront of strategies underlying the next generation of crop protection measures.

Global RNA profiling and GO term enrichment highlighted specific genes and biological processes to target using RNAi and provided useful selection criteria for TIP. For example, suppressing transcripts involved in cell wall modification (e.g. SS1G_12021) and fungal ROS response proteins (e.g. SS1G_05899, SS1G_02495) impeded fungal establishment on the leaf surface by interfering with the maintenance of structural integrity and the protection of the advancing fungal hyphae from plant respiratory ROS defense^43,44^. Furthermore, by identifying homologues of essential genes from the model organism *A*. *fumatigus*^45^, despite the overall processes being downregulated during infection, flexibility was added to the TIP-guided selection for dsRNA control. The TIP protocol improves upon previous topical implementations of RNAi biotechnology, which relied on transgenic approaches for its development^46,47^. Together, the selection criteria developed offers the first guide for phytopathogenic fungal management and demonstrates a definitive link for the application of large scale data and fungal control.

During infection, *S*. *sclerotiorum* secretes an arsenal of pathogenicity factors, including hydrolases and nutrient acquisition enzymes, which form a resilient genetic system to regulate metabolic homeostasis^48–50^. Consequently, when targeting upregulated processes during infection, such as carbohydrate catabolism (e.g. SS1G_00509), cell wall degrading enzymes (e.g. SS1G_04551) and hydrolase activity (e.g. SS1G_13982), no significant impacts on lesion sizes were observed. Similarly, a high level of gene regulation may also reduce the effect of RNAi-based control. For example, the essential pathogenicity factor *Ssoah* (SS1G_08218; oxaloacetate acetylhydrolase), is known to be heavily regulated by *SsPac1*^51^. Thus, the RNAi-induced reduction in *Ssoah* transcript level may be rescued by the intrinsic regulatory network responsible for *S*. *sclerotiorum* pathogenesis. Other factors such as a prolonged protein half-life, or negative pathogenicity regulation, already observed in *S*. *sclerotiorum*, could also fail to elicit plant protection despite successful transcript knockdown^52,53^. Thus, by screening a great variety of genes sharing single biological processes, poor targets can be excluded from the RNAi target selection.

Transcript accumulation levels may have affected the efficiency of RNAi silencing in *S*. *sclerotiorum* and were therefore an important consideration for the construction of TIP. For the transcripts SS1G_13720, SS1G_06055, and SS1G_13982, which accumulated at low levels under all conditions, no significant changes in lesion formation were observed with specific dsRNA treatment. Previously, low transcript accumulation levels resulted in poor siRNA-mediated knockdown in human cells, suggesting that a threshold level of target mRNA must be present for the activation of the RISC complexes^54,55^. In contrast, transcripts of the genes *Ssoah* and SS1G_10167 accumulated at high levels under all conditions, also failed to respond to the administered dsRNA in the infection assays. In other organisms, genes with high transcription rates have been difficult to knock down using RNAi, presumably because the dose of dsRNA was insufficient to eliminate enough of the target transcripts^37,56^. At the dsRNA dose tested, most lesion size reductions were observed with dsRNAs that targeted moderately expressed transcripts (10–500 FPKM). Genes that showed large deviations in expression levels in the infected plants, relative to the *in vitro-*grown fungus, were not significantly affected by the dsRNA treatments. RNA-seq is a powerful tool to discover drastic transcript fold-changes caused by stronger promoter induction and activity under infection conditions^57,58^. By incorporating RNA-seq experiments into effective dsRNA molecule design^59^, highly induced genes can be avoided, and genes with moderate upregulation during infection can be preferentially selected.

The protection imparted by the dsRNA molecules on both *B*. *napus* and the related crucifer *A*. *thaliana*, suggests a common infection strategy employed by *S*. *sclerotiorum*, making topical application of dsRNAs an attractive option for fighting fungal pathogens with extensive host ranges. The differences in the extent of protection may be attributed to variations in leaf architecture, such as larger cell size, cell wall, and thicker cuticle of *B*. *napus*, which can structurally limit fungal growth^60–62^. Moreover, dsRNAs targeting *B*. *cinerea* DCL1 and 2, previously mitigated infections on a variety of produce and horticultural tissues^16^. Therefore, topical dsRNA designs could also prove useful during post-harvest storage, resisting fungal damage in transport and on store shelves. Taken together, the array of dsRNAs that produced strong transcript knockdown and lesion size reductions invites future studies designed to optimize formulations to translate the success into agronomic solutions.

Our experiments demonstrated that dsRNA targeting a single transcript applied to the leaf surface suppressed lesion growth in *S*. *sclerotiorum* and *B*. *cinerea*. A recent report by Wang *et al*.^16^ showed that dsRNAs targeting transcripts encoding both DCL 1 and 2, were able to suppress *B*. *cinerea* and *Verticillium dahliae* infection of transgenic *A*. *thaliana* containing a construct to produce dsRNA molecules targeting both species^16^. While most of the tested dsRNAs of *S*. *sclerotiorum* and *B*. *cinerea* reduced disease symptoms significantly in *B*. *napus*, some targets showed greater reductions in disease severity. Both pathogens likely share a common cluster of genes to control pathogenicity in a broad range of host plant species, however *B*. *cinerea* likely operates through alternative pathogenic pathways to control infection in different host plants^63,64^. The moderate correlation between the efficacy of RNAi targets in the two fungi tested suggests that homologues could be an initial step for identifying targets, which could be later fine-tuned using TIP, for broad levels of fungal control. Thus, additional target discovery of any host-pathogen system is warranted to identify key targets for RNAi-based control of fungal pathogens.

RNA-seq technology offers comprehensive insights into the genes and processes involved in fungal pathogenesis and is an ideal starting point for designing RNAi-based management strategies. We present a flexible structure for identifying alternative target transcripts, such that topical applications of dsRNA can be extended to control a variety of fungal pathogens and protect other agronomically important plant species. The target identification pipeline provides a new, adaptable platform for the design of RNAi biotechnology and marks a substantial evolution of next-generation fungal phytopathogenic control. Further examinations of pathosystems will identify additional efficient RNAi targets across multiple fungi to improve broad fungal control.

## Methods

### *Brassica napus* growth conditions

Seeds of *B*. *napus* cv. Westar and *B*. *napus* cv. ZY821 were grown in Sunshine Mix No. 1 (SunGro Horticulture, Agawam, MA, USA) at 22 °C and 50–70% humidity under long day conditions (16 hours light, 8 hours dark 150–200 µE/m^2^/s). ZY821 plants were subjected to a 1-month vernalization treatment after planting (8 hours light, 16 hours dark, 8–10 °C, 40% humidity and 100 µE/m^2^/s), before being transferred back to long day conditions. The plants were grown until 30% bloom stage for use in experiments^28^.

### Leaf inoculation and *S*. *sclerotiorum* tissue collection for RNA sequencing

*Sclerotinia sclerotiorum* cultures were collected at the Morden Research and Development Centre, Agriculture and Agri-Food Canada, Morden, MB, Canada. Ascospores were generated from sclerotia that were germinated carpogenically using specialized medium (54 g cornmeal, 3.5 g vermiculite, 37.5 mL of a 1% casamino acids and 1% yeast extract solution), and incubation on wet sand at 20 °C to induce apothecia^65,66^. Once generated, ascospores were stored on tin foil at 4 °C in desiccant in the dark. *Sclerotinia sclerotiorum* ascospores (8 × 10^4^ mL^−1^) were suspended in a 0.02% Tween 80 (Sigma-Aldrich, St. Louis, MO, USA) solution. 25 µL of the ascospore solution was transferred onto senescing *B*. *napus* petals in a petri plate and sealed with Parafilm. Ascsospore-inoculated petals were stored at room temperature (21 °C) for 72 hours and allowed to germinate prior to being inoculated on the leaf surface.

*Brassica napus* petals were then transferred to cv. Westar and ZY821 leaves between 1–3 PM at the 30% bloom stage and covered with a clear plastic bag to maintain high relative humidity. After 24 hours, at least ten lesions (1 cm^2^) were collected from the site of inoculation for each of three biological replicates to identify early infection stage pathogenicity factors. To identify genes associated with *S*. *sclerotiorum* grown *in-vitro*, sclerotia were cut into halves and placed open side down on PDA media (Difco Laboratories Inc., Detroit, MI, USA)^67^. Mycelium tissue was collected from the leading hyphal edge after 3 days and flash frozen in liquid nitrogen prior to RNA isolation.

### RNA extraction and sequencing

RNA was isolated using Invitrogen Plant RNA Purification Reagent and treated with Ambion Turbo DNA-free DNase according to the manufacturer’s protocol (Thermo Fisher, Waltham, MA, USA). Quantity and purity were assessed spectrophotometrically and the quality of RNA samples verified using electropherogram profiles and RNA Integrity Numbers (RIN) with an Agilent 2100 Bioanalyzer and RNA Nano Chip (Aligent, Santa Clara, CA, USA). RNA-Sequencing cDNA libraries were prepared from 5 µg of total RNA according to the methods of Kumar *et al*.^68^ with some modifications. Isolation of mRNA from was performed using the NEBNext® Poly(A) mRNA Magnetic Isolation Module (New England Biolabs, Ipswich, MA, US) according to manufacturer’s instructions with the following modifications: all reaction volumes were 7.5 µL of Oligo d(T)_25_ beads per sample. The remaining preparation steps were performed according to the HTR protocol starting with the first strand cDNA synthesis. NEXTflex™ ChIP-Seq Barcodes (Bio Scientific, Austin, TX, USA) were used as adaptors for the adapter ligations and NEXTflex™ PCR Primer Mix for the library enrichment PCR step. Library quality was assessed using a High Sensitivity DNA chip on an Agilent 2100 Bioanalyzer and size selected using E-Gel® SizeSelect™ 2% agarose gel (Life Technologies, Carlsbad, CA, USA) to target fragments from 250–500 bp in length. 100 bp single-end RNA sequencing was carried out using the Illumina HiSeq. 2000 platform (Génome Québec Innovation Centre, McGill University, Montreal, Canada).

### Bioinformatics Pipeline

FastQ files were trimmed using Trimmomatic 0.33^69^: adapter sequences, initial 12 bases of raw reads, low quality reads with a quality score under 20 over a sliding window of 4 bases, and reads with an average quality score under 30 removed during the trimming process. Remaining reads shorter than 50 nucleotides were also removed. The splice junction mapping software TopHat (v2.1.0, http://ccb.jhu.edu/software/tophat/index.shtml)^70^ was then used to align trimmed reads to the *S*. *sclerotiorum* genome^63^. Gene expression was quantified using Cuffquant (v2.2.1, https://github.com/cole-trapnell- lab/cufflinks)^70^ and expression values normalized to FPKM using Cuffnorm (v2.2.1, https://github.com/cole-trapnell-lab/cufflinks)^70^. Differential expression analysis was performed using Cuffdiff (v2.2.1, https://github.com/cole-trapnell-lab/cufflinks) and resulting significantly differentially expressed genes used as input for GO term enrichment using SeqEnrich (https://github.com/nagreme/SeqEnrich)^71^. GO terms were collected from UniProt KB (http://www.uniprot.org/)^72^ and kindly made available by Nicolas Lapalu (L’Institut national de la recherché argonomique, Versailles, France)^63^. Clustering analyses were performed using the pvclust package in R studio (https://cran.r-project.org/web/packages/pvclust/index.html) for hierarchical clustering and the DESeq package in R studio for principle component analysis clustering, in both analyses the Cuffnorm outputted FPKM transcript expression values with a value >1 were used as input values for clustering (http://www-huber.embl.de/users/anders/DESeq/)^73^. GO term heat map visualization was carried out using the gplots package in R studio (https://cran.r-project.org/web/packages/gplots/index.html), terms were considered enriched at *P* < 0.001 with a blue-red color scale representing levels of statistical enrichment. Venn diagram visualization was performed using Venny 2.1 (http://bioinfogp.cnb.csic.es/tools/venny/)^74^.

### Selection of gene targets and Target Identification Pipeline (TIP)

Targets for RNAi were identified from a list of differentially upregulated genes shared between *S*. *sclerotiorum* grown on Westar and ZY821 compared to *in vitro* control. Essential genes from close relatives were also identified using the Database of Essential Genes (www.essentialgene.org)^45^ and known regulators of infection^29^.

Genes were then selected based on enrichment of biological processes (GO terms) associated with cell wall modification, mitochondria, ROS response, protein modification, pathogenicity factors, transcription, splicing, protein modification, and translation while those associated with growth, transport, transcription factors, electron carriers, signal transduction, pigment synthesis, and carbohydrate metabolism were avoided due to the complex nature, functional redundancy, and non-compromising roles the biological process at play. Putative functions and accessions were determined and confirmed using NCBI (National Center for Biotechnology Information; http://www.ncbi.nlm.nih.gov/) and KEGG (Kyoto Encyclopedia of Genea and Genomes; http://www.genome.jp/kegg/)^75^. Only targets of at least 200 nucleotides were selected to avoid natural sequence variations that could impair RNAi silencing^42^.

The list of target genes was reduced further based on FPKM values of 1–500 and log2-fold change thresholds of −0.5 and 4. Highly regulated targets, targets with functional redundancy and genes with multiple homologues were also avoided. Genes encoded within organelles, such as mitochondria were also avoided since mitochondria cannot process or import the dsRNA from the cytoplasm. The Target Identification Pipeline is summarized in Table S2.

### RNA extraction, cDNA synthesis and *in vitro* production of dsRNAs

Actively growing fungal hyphae grown *in vitro* were ground in liquid nitrogen and RNA extracted using Invitrogen Plant RNA Reagent (Invitrogen, Carlsbad, CA, US) and treated with Turbo DNase (Ambion, Carlsbad, CA, US). cDNA was synthesized with the Maxima First Strand reverse transcriptase (Thermo Scientific, Waltham, MA, US) using 500 ng of RNA in a 10 μL reaction.

*Sclerotinia sclerotiorum* and *B*. *cinerea* gene sequences (Genbank; http://www.ncbi.nlm.nih.gov/) and primers were designed using Primer BLAST (www.ncbi.nlm.nih.gov/tools/primer-blast) to PCR amplify gene fragments ranging between 200 and 450 bp in length and quality assessed using Primer3 (http://bioinfo.ut.ee/primer3/)^76^. Primer sets were designed to limit regions of homology (>20 bases) to other Eukaryotes by searching BLASTN (http://blast.ncbi.nlm.nih.gov/Blast) RefSeq accessions to discover sequence homologies in putative dsRNA. A BLASTN query using *Sclerotinia sclerotiorum UF-80* RefSeq entries was performed to ensure each dsRNA molecule reacted with a single transcript within the fungus^77^. A complete list of primers used in the paper are found in Additional file 3.

Target gene sequences were amplified using Phusion Taq (Thermo Scientific, Waltham, MA, US) under the following conditions: 98 °C for 30 s; 35 cycles of: 98 °C for 10 s, 57 °C for 20 s, and 72 °C for 20 s; and a final extension of 72 °C for 7 min. Amplicons were gel purified (New England Biolabs, Ipswich, MA, US) and digested using FastDigest KpnI and XbaI or XhoI (Thermo Scientific, Waltham, MA, US) according to the manufacturer’s protocols. The products were ligated into the similary digested pL4440 vector (kindly donated by Andrew Fire, Stanford University) using T4 ligase (Invritogen, Carlsbad, CA, US) according to the manufacturer’s protocol. Prepared plasmids were used to transformed *E*. *coli* MachI cells (Thermo Scientific, Waltham, MA, US) and sequence inserts were confirmed using Sanger Sequencing (The Centre for Applied Genomics. Toronto, ON, Canada).

Primers (F: CAACCTGGCTTATCGAA; R: TAAAACGACGGCCAGTGA) designed to amplify T7 promoters flanking each insert were used in a Phusion PCR: 98 °C for 3 min, 35 cycles of: 98 °C for 15 s, 57 °C for 15 s, and 72 °C for 40s; and a final extension of 72 °C for 10 min. The PCR reaction was purified using a PCR cleanup kit (New England Biolabs, Ipswich, MA, US) and dsRNA synthesized using the MEGAScript^TM^ RNAi kit (Invitrogen, Carlsbad, CA, US) according to manufacturer’s instructions.

### Quantification of relative transcript abundance following dsRNA application *in vitro*

A 1 mm plug was taken from the leading edge of freshly cultured 3-day old fungal colony and placed in stationary 7 mL of potato dextrose broth (Difco Laboratories Inc., Detroit, MI, USA) containing ampicillin (50 μg/mL; MP Biomedicals Inc., Santa Ana, CA, USA) in a 60 mm × 15 mm petri dish for 48 hours. DsRNAs were applied at a dose of 500 ng mL^−1^ and tissue collected at 0, 24, 48, 72, and 96 hpi. To examine the effect of dsRNA concentration on target gene knockdown, 100 ng ml^−1^, 200 ng ml^−1^, 500 ng ml^−1^, and 1000 ng ml^−1^ of dsRNA were added to a 3 mL liquid medium, shaking at 200 rpm, and tissue collected 3 dpi.

Transcript levels for the target genes were determined using qPCR on the Bio-Rad CFX96 Connect Real-Time system using SsoFast EvaGreen Supermix (Bio-Rad Laboratories, Hercules, CA, US) in 10 μL reactions according to the manufacturer’s protocol under the following conditions: 95 °C for 30 s, and 45 cycles of: 95 °C for 2 s and 60 °C for 5 s. Melt curves with a range of 65–95 °C with 0.5 °C increments were used to assess nonspecific amplification, primer dimers, and aberrant amplifications. Primers and corresponding efficiencies are given in Additional file 3. Relative accumulation was calculated using the ΔΔCq method, normalized to Sac7 (SS1G_12350) and relative to *GFP*-dsRNA control with the corresponding dose^78,79^.

### Foliar applications of dsRNAs to the leaf surface

Senescing petals of *B*. *napus cv*. *Westar* were inoculated with 20 ng μL^−1^ dsRNA or water, 0.015% Silwet L-77 (Lehle Seeds, Round Rock, TX, US), and 10 μL of *S*. *sclerotiorum* spores in water (5 × 10^5^ spores ml^−1^). The petals incubated for 3 days^28^. After, a 25 μL solution of 200 ng dsRNA or water and 0.03% Silwet L-77 was applied to the leaf surface of approximately plants at the 30–50% flowering stage (n = 10). The application was allowed to dry (approximately 15 min) before a senescing petal was applied to the same spot and allowed to incubate under high humidity for 2 days. A total of 59 *S*. *sclerotiorum* genes targets were selected from (i) the Database of Essential Genes (DEG)^45^, (ii) literature searches^29^, and (iii) the Target Identification Pipeline (TIP) (Table S2). TIP genes were nominated based on a range of selection criteria, including: common significant expression within the RNA-Seq dataset; biological function; moderate expression levels (between 10 and 500 FPKM); fold changes between −0.5 and 4 (infection relative to *in vitro*); and biological processes summarized in Table S2. Petals were pre-treated with dsRNA and *S*. *sclerotiorum*-colonized petals were then placed over the dsRNA-treated leaf surfaces. Fungal lesion size was scored 2 dpi.

For the Arabidopsis assays, 25 day old leaves were treated with 10 μL of 200 ng of dsRNA and 0.02% Silwet L-77 and allowed to dry. A 10 μL *S*. *sclerotiorum* spore solution (5.5 mM glucose, 62.5 mM KH_2_PO_4_ (Sigma Life Science, St. Louis, MO, US); 1 × 10^6^ spores mL^−1^ was spotted on the surfaces of the dsRNA coated leaves (n = 30) and allowed to incubate under high humidity for 4 days^30^.

For *B*. *cinerea* assays, a 12 μL solution containing 500 ng of dsRNA and 0.03% Silwet L-77 was applied to the leaf surface of *B*. *napus* cv. Westar. Following a complete drying period, 10 μL of buffered *B*. *cinera* spores (5.5 mM glucose, 62.5 mM KH_2_PO_4_; 1 × 10^5^ spores mL^−1^) were placed on the same spot (n = 40) and allowed to incubate for 4 days^30^. In all cases, lesion size was quantified using ImageJ software (imagej.nih.gov). Water and *GFP*-dsRNA were both used as controls during the foliar assays and neither were significantly different from each other (Figure S2; student’s t test; p = 0.6).

### Statistical analysis

To analyze the data, JASP (jasp-stats.org) statistical software was used to compute hypothesis testing^80^. To test the effect of timing and dosing of dsRNA treatment on the relative mRNA accumulation, data were subjected to a one-way analysis of variance (ANOVA with p < 0.05), followed by a comparison of means using a Tukey post hoc test with significance levels set at p < 0.05^81^. To determine whether dsRNA treatment on the surface of the leaf differed from control, *student’s t-tests* were performed with a Bonferroni correction to the level of significance. Correlation was calculated using Pearson’s correlation.

## Electronic supplementary material

Supplementary Figures and Table

dsRNA foliar results

Primer sequences

RNA seq bioinformatics
