# Supplementary material for: Identification and application of exogenous dsRNA confers plant protection against Sclerotinia sclerotiorum and Botrytis cinerea
Source: Sci Rep. 2018 May 9;8:7320. doi: 10.1038/s41598-018-25434-4 (PMC5943259; doi:10.1038/s41598-018-25434-4)
Supplement: Supplementary file 1 — Supplementary Figures and Table [file 41598_2018_25434_MOESM1_ESM.pdf]

Supplementary file

Identification and application of exogenous dsRNA confers plant protection against *Sclerotinia sclerotiorum* and *Botrytis cinerea*

Austein G Mcloughlin, Nick Wytinck, Philip L Walker, Ian J Girard, Khalid Y. Rashid, Teresa de Kievit, WG Dilantha Fernando, Steve Whyard, and Mark F Belmonte

Table S1. Mapping summary for all biological replicates of *in-vitro* and *in-planta* samples. Raw RNA-sequencing reads trimmed using Trimmomatic 0.33 and aligned to the *S. sclerotiorum* genome using TopHat v2.1.0.

	in-vitro (1)	in-vitro (2)	in-vitro (3)	ZY821 24hpi (1)	ZY821 24hpi (2)	ZY821 24hpi (3)	Westar 24hpi (1)	Westar 24hpi (2)	Westar 24hpi (3)
**Trimmed reads**	6537111	6339049	11826235	31473436	44428372	20453088	40287633	37020385	52025758
**Mapped reads**	5388394	5216455	10273216	6282525	6724081	3061161	7810054	6478810	8125640
**% Mapped**	82.4	82.3	86.9	20	15.1	15	19.4	17.5	15.6

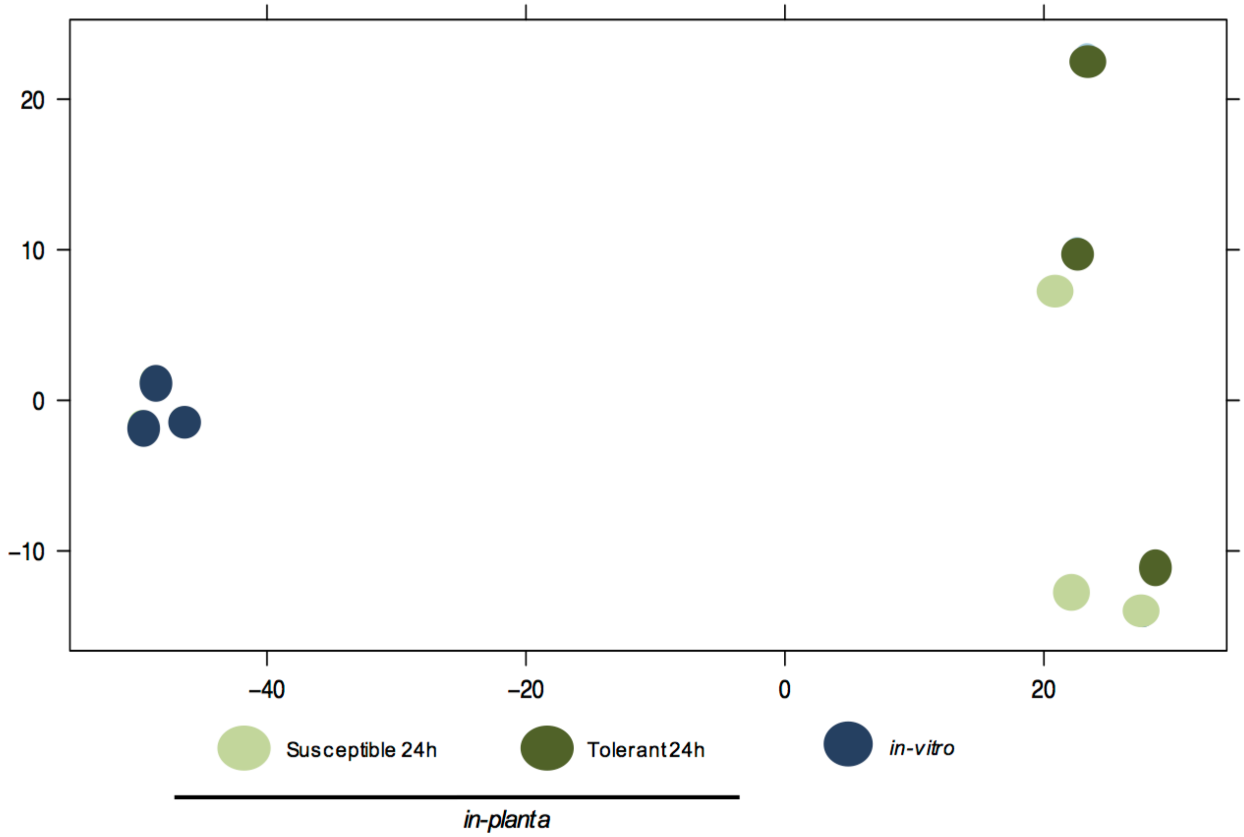

Figure S1. *Principle component analysis plot of in-planta and in-vitro grown S. sclerotiorum based on FPKM transcript expression.*

Table S2. Target identification pipeline guidelines for selecting targets to control phytopathogenic fungi using RNAi

Criteria	Choose	Avoid
**RNA-seq dataset**	Upregulated genes common amongst infection conditions	Down regulated genes in response to infection
**Essential Genes**	Genes of closely related species. Genes can be found in the Database of Essential Genes [1]	Essential genes from distant or unrelated relatives
**Biological processes**	Mitochondria, ROS response, protein modification, pathogenicity factors, transcription, splicing, protein modification, translation, cell wall modification	General growth, transcription factors, transport, electron carriers, signal transduction, pigment synthesis, carbohydrate metabolism
**Expression levels**	FPKM values between 1 and 500	Lowly (FPKM <1) and Highly (FPKM > 500) expressed during infection
**Log2-fold change (control vs. infection)**	Fold changes between -0.5 and 4	Fold changes below -0.5 and above 4
**Transcript length**	Above 200 nucleotides	Below 200 nucleotides
**Gene location**	Nuclear-encoded	Organelle-encoded
**Redundancy**	Single function or without homologues	Genes with multiple homologues and functionally similar roles
**Regulation**		Genes that are heavily regulated

(**1**) www.essentialgene.org [1]

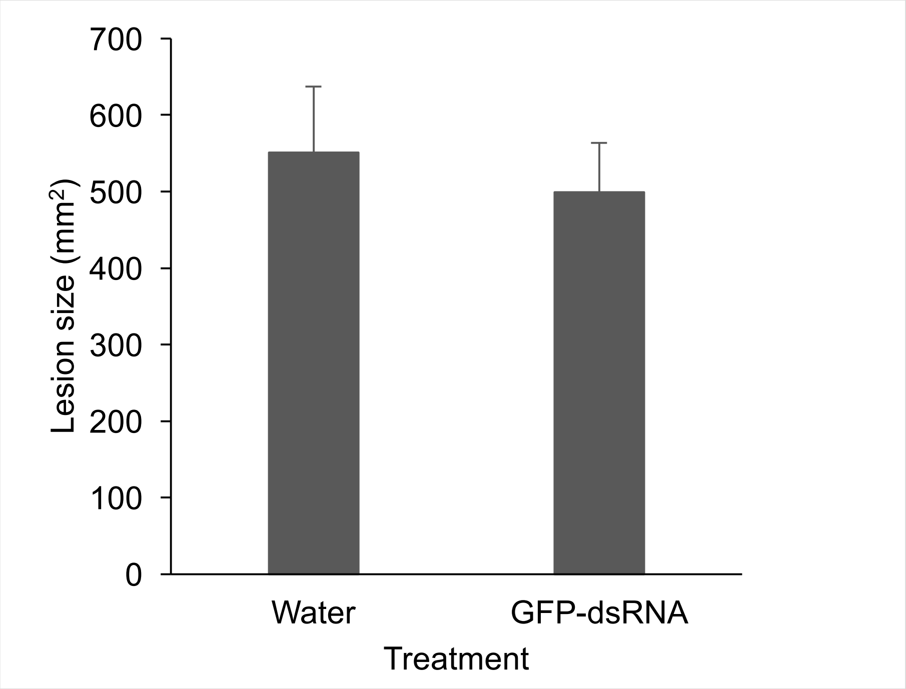

Figure S2 *Sclerotinia sclerotiorum infection on B. napus leaves (n = 10) 2 dpi after treatment of water or GFP-dsRNA. No significant difference was observed (p = 0.63, student's t-test).*

References

1. Luo, H., Lin, Y., Gao, F., Zhang, C. T. & Zhang, R. DEG 10, an update of the database of essential genes that includes both protein-coding genes and noncoding genomic elements. *Nucleic Acids Res.* **42,** 574–580 (2014).
